# Supplementary material for: Epithelial Sodium Channel Alpha Subunit (αENaC) Is Associated with Inverse Salt Sensitivity of Blood Pressure
Source: Biomedicines. 2022 Apr 23;10(5):981. doi: 10.3390/biomedicines10050981 (PMC9138231; doi:10.3390/biomedicines10050981)
Supplement: Supplementary file 1 [file biomedicines-10-00981-s001.zip › biomedicines-1623455-supplementary.pdf]

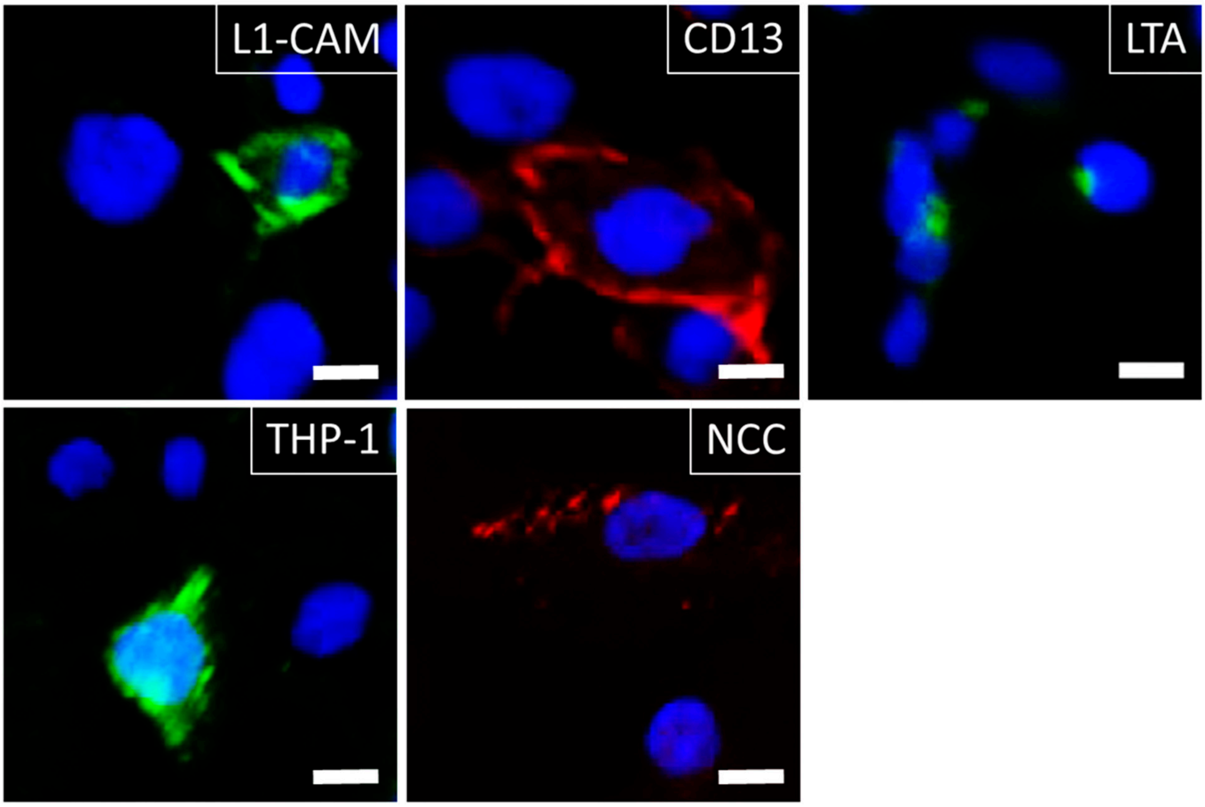


**Supplemental Figure S1**: Representative images show different types of renal cells in the urine. L1-Cam is collecting duct cell (CDC) marker, CD13 and LTA are proximal tubule cell (PTC) markers. THP is a thick ascending limb marker. NCC is distal tubule cell marker. Scale bar=10 µm

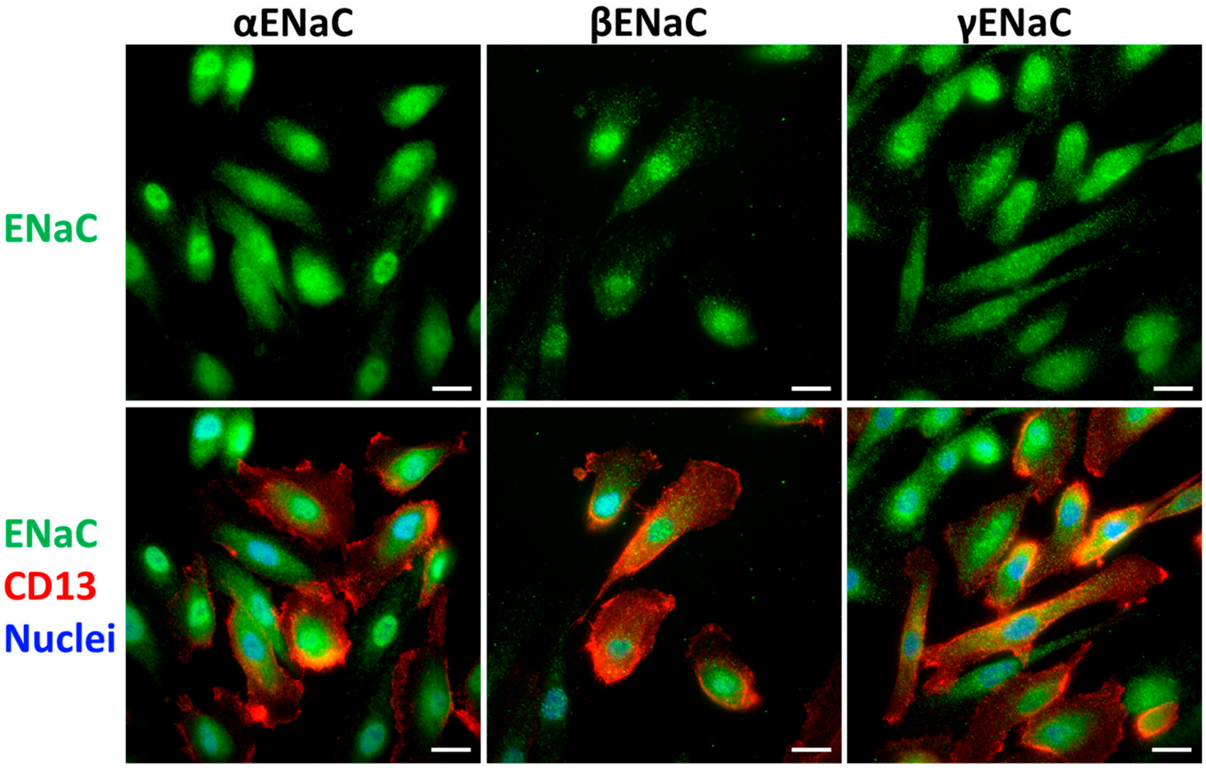


**Supplemental Figure S2:** $\alpha$ENaC, βENaC and γENaC staining in permeabilized urine-derived renal tubule cells. Nuclei are in blue. $\alpha$ENaC, βENaC and γENaC are in green. CD13 is in red. Scale bar =10 μm

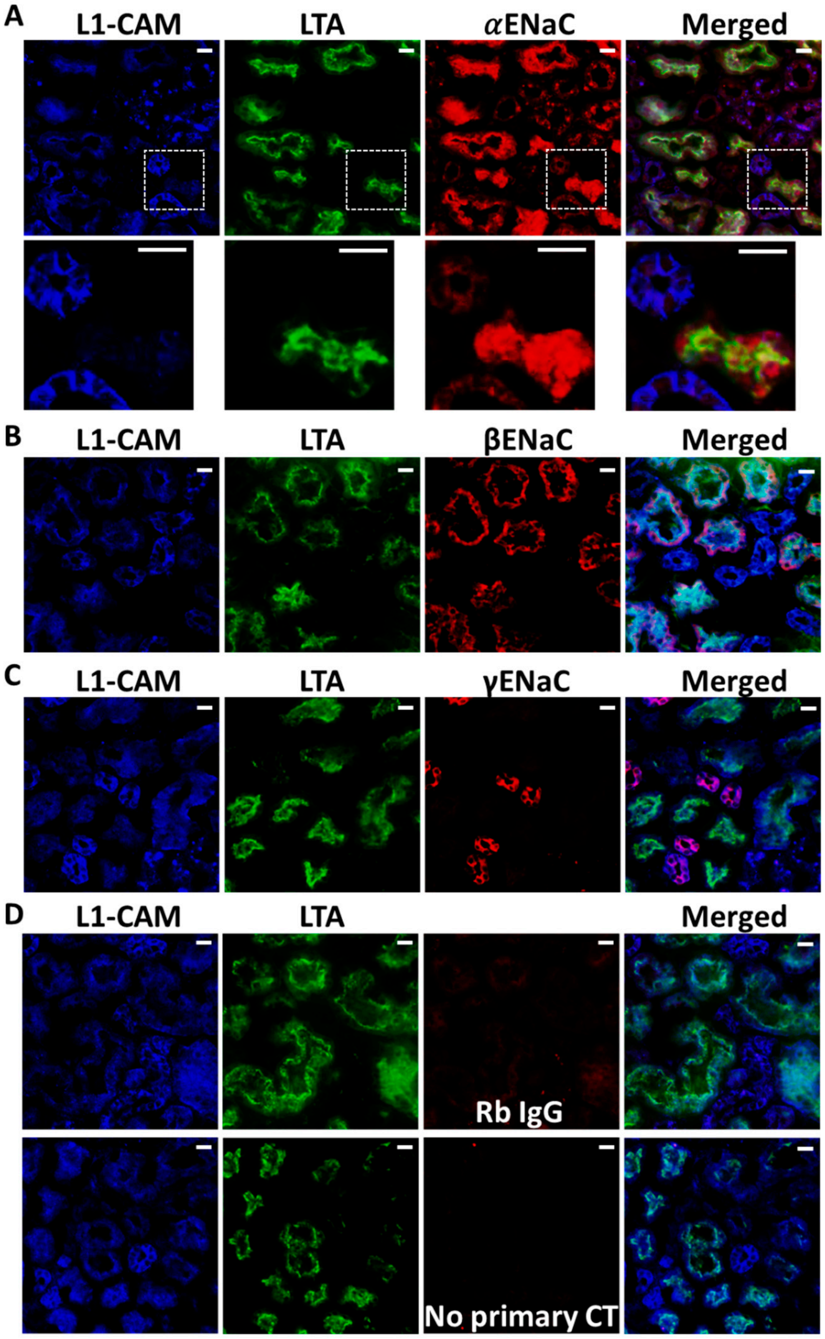


**Supplemental Figure S3**. αENaC, βENaC and γENaC (StressMarq antibodies) staining in human renal cortex. Both αENaC and βENaC are present in PTC. γENaC is restricted within CDCs. αENaC, βENaC and γENaC are in red. LTA, a PTC marker, is in green. L1-CAM, a CDC marker, is in blue. Rb (rabbit) IgG and no primary antibody are tested as negative controls. The lower panel of (A) is zoomed-in views of boxed regions of the upper panel. Scale bar = 10 µm

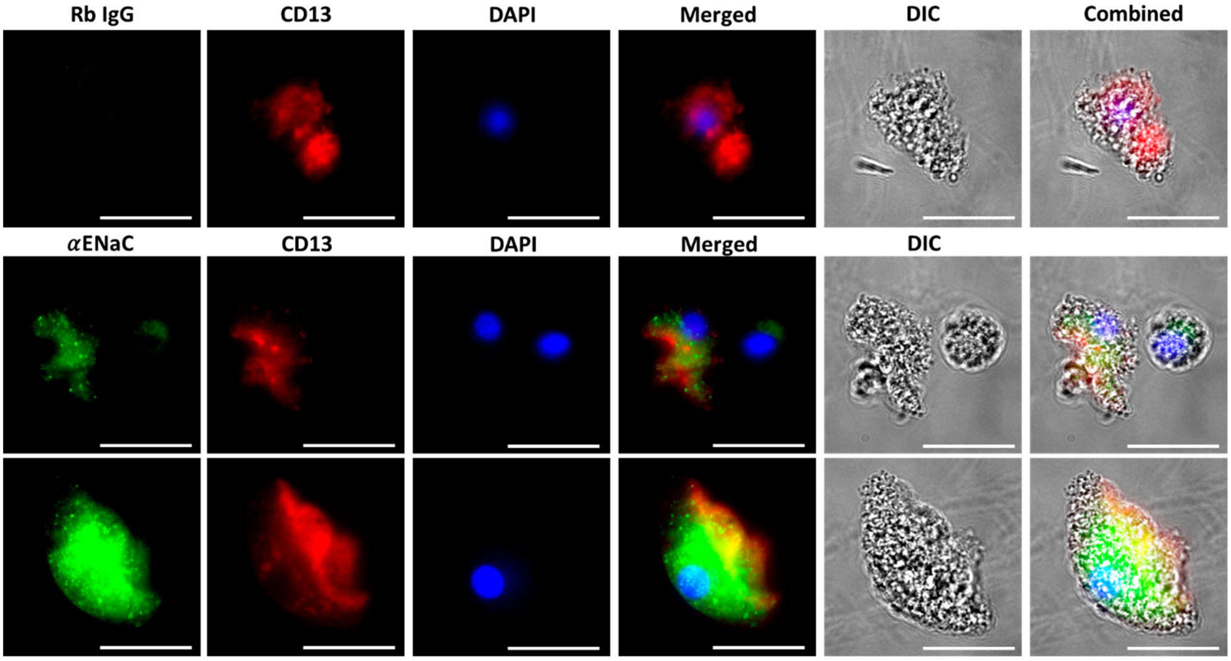


**Supplemental Figure S4**: $\alpha$ENaC was stained in cells digested from fresh human renal cortex in suspension. Rabbit IgG was used for negative control. CD13 positive cells also shows strong $\alpha$ENaC staining on their apical side. Scale bar = 10 µm

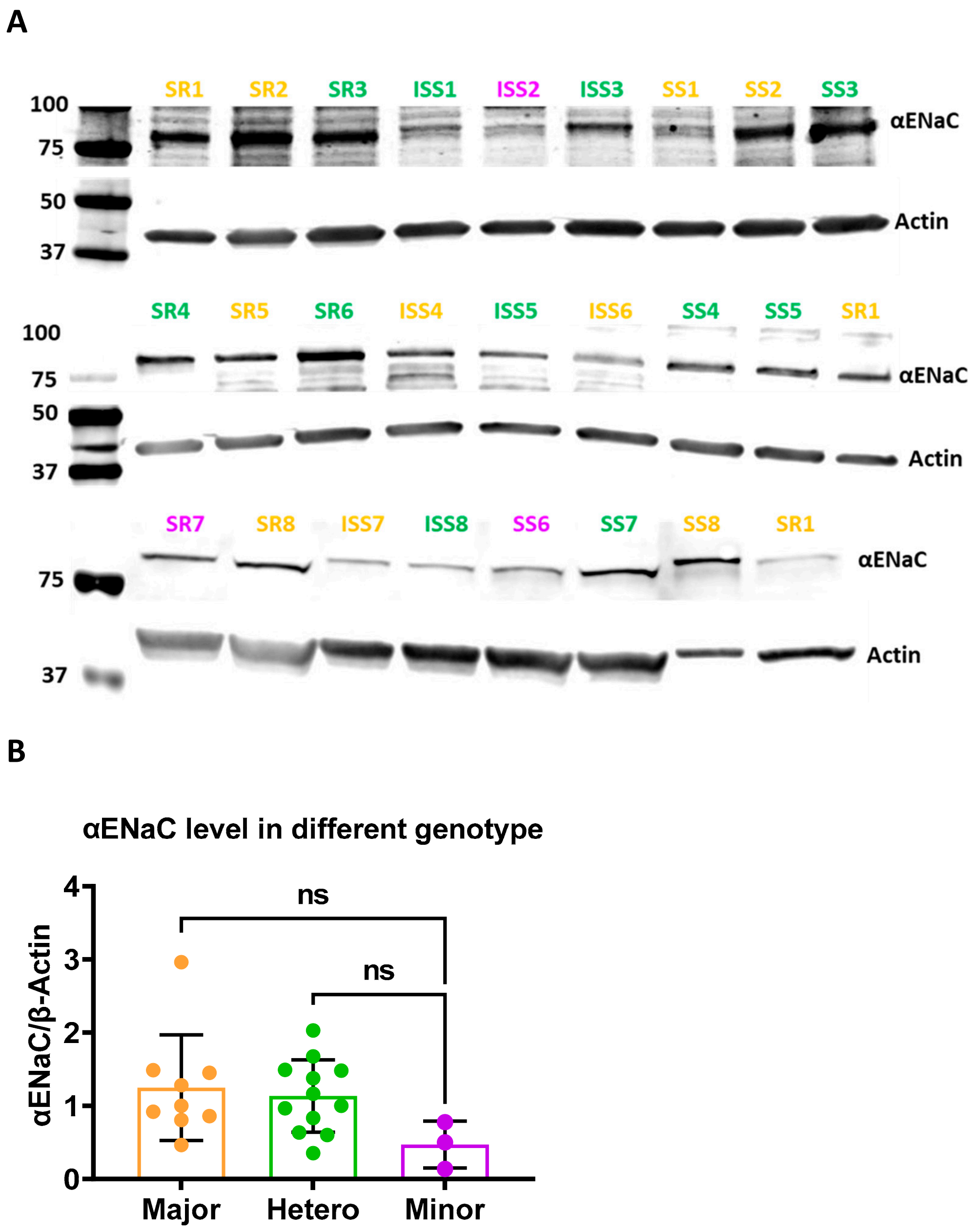


**Supplemental Figure S5**. αENaC western blot analysis in more urine-derived hRTC lines. (A) αENaC western blot in another 15 urine-derived hRTC lines. SR, salt resistant, SS salt sensitive; ISS inverse salt sensitive. The calculation was normalized by SR1 on each blot. (B) αENaC level in different genotype. Homozygous major variants are in orange; heterozygous samples are in green; homozygous minor variants are in purple.

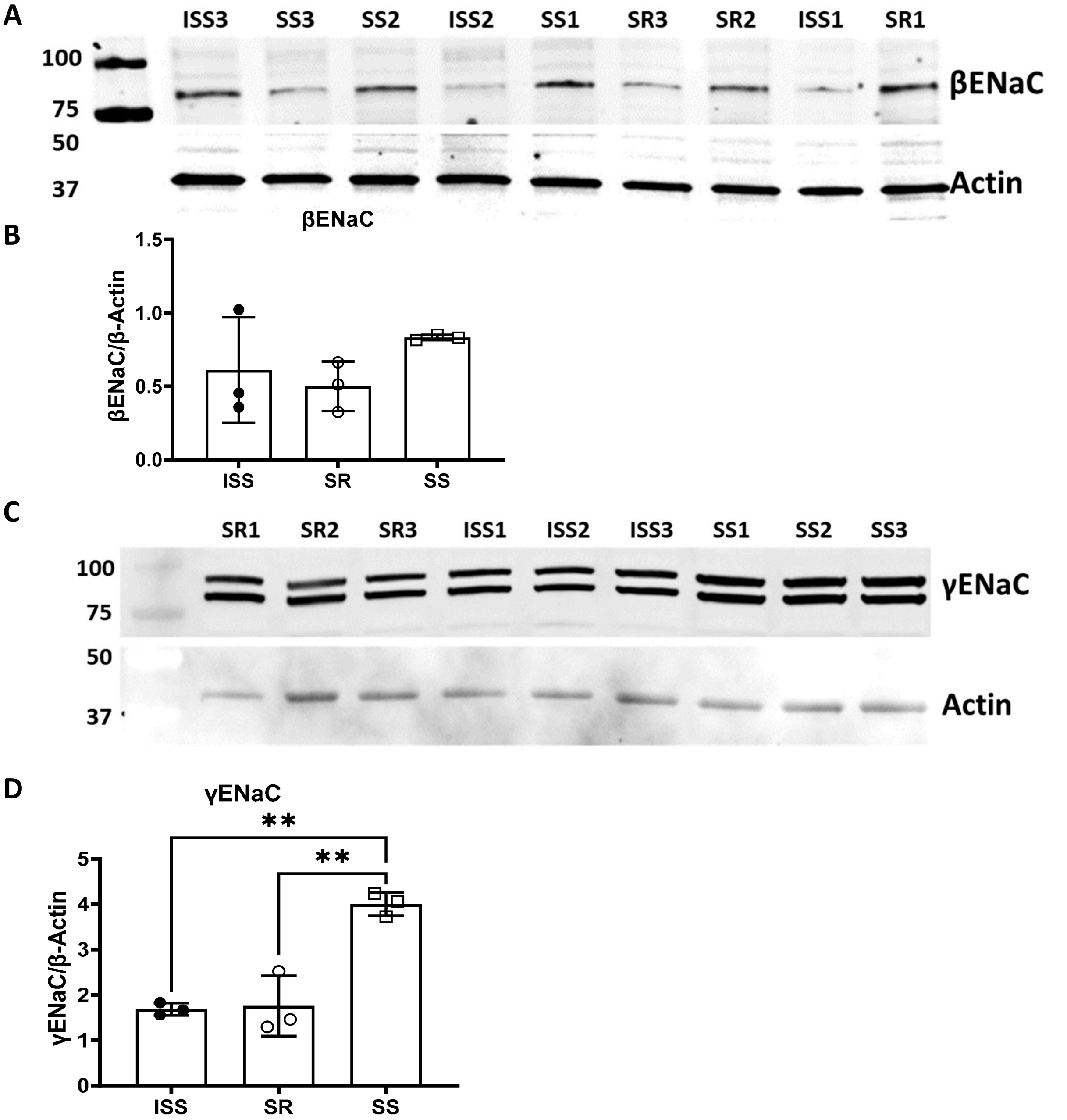


**Supplemental Figure S6**: βENaC and γENaC are found in CD13⁺ urine-derived hRTCs. Actin was used as loading control in all western blot analysis. A and B) There is no significant difference of βENaC among different phenotypes. C and D) γENaC is significantly higher in SS than both SR and ISS.

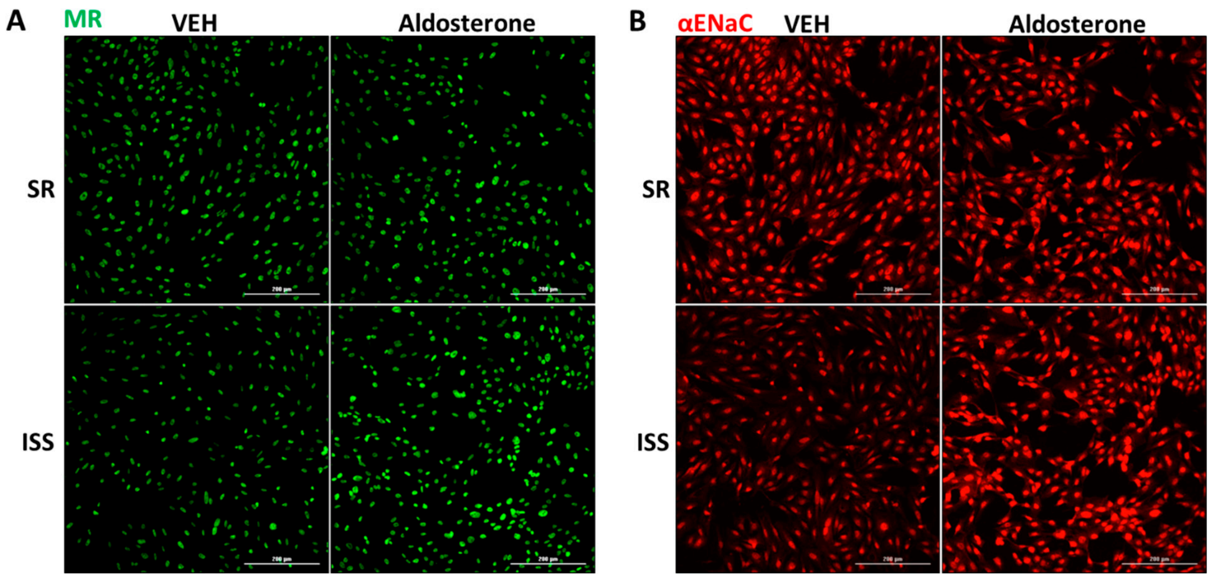


**Supplemental Figure S7**. Representative images of mineralocorticoid receptor (MR) and αENaC immunostaining under aldosterone treatment. MR staining is in green (A), and αENaC is stained in red (B). Scale bar = 200 µm

**Supplemental Table S1**

**Characteristics of UVA Study Subjects (Mean±SD)**

| | **Inverse Salt Sensitive Subjects** | **Salt Resistant Subjects** | **Salt Sensitive Subjects** | **P Value** |
|---|---|---|---|---|
| Number of subjects | 37 | 178 | 65 | |
| Male | 12 | 78 | 23 | 0.2837[+] |
| Female | 25 | 100 | 42 | |
| Age (yrs) | 40.8±15.2 | 42±15.1 | 50.5±14.2 | 0.0002 |
| BMI | 25.1±3.0 | 24.8±2.9 | 25.3±2.9 | 0.4716 |
| **<u>After Low Sat Diet</u>** | | | | |
| Systolic (mmHg) | 121±13.3 | 117.2±13.8 | 114.9±12.7 | 0.0915 |
| Diastolic (mmHg) | 76.2±8.4 | 72.9±9.1 | 71.7±6.5 | 0.0345 |
| MAP (mmHg) | 91.1±9.5 | 87.7±10 | 86.1±7.8 | 0.0382 |
| **<u>After High Sat Diet</u>** | | | | |
| Systolic (mmHg) | 110.3±12.2 | 118.5±14.1 | 129.5±12.9 | <0.0001 |
| Diastolic (mmHg) | 66±7.3 | 71.9±9.6 | 80±8.2 | <0.0001 |
| MAP (mmHg) | 80.7±8.6 | 87.4±10.5 | 87.4±10.5 | 0.0013 |

For the continuous variables, one-way ANOVA was used to compare the three groups.

+ Comparison of distribution of gender in three groups was done by Chi-square test.

**Supplemental Table S2: Odds Ratio**

| | ISS | NON-ISS (SR+SS) |
|---|---|---|
| **SNPed (Homozygous minor variant and heterozygous)** | 23 | 112 |
| **NON-SNPed (homozygous major variant)** | 14 | 130 |

Odds ratio = 23*130/(14*112)=1.91, indicating the odds are 1.91 times higher that people who have rs4764586 will become ISS compare to those who are homozygous major variant.
